# Primary Intraosseous Synovial Sarcoma in the Mandible

**DOI:** 10.1155/2021/9945591

**Published:** 2021-11-28

**Authors:** Lucas Novaes Teixeira, Eduardo Zambaldi da Cruz, Ana Cláudia Garcia Rosa, Anderson Abdo Rodrigues, Fabrício Passador-Santos, Vera Cavalcanti de Araújo, Andresa Borges Soares

**Affiliations:** ^1^Faculdade São Leopoldo Mandic, Rua José Rocha Junqueira 13, Swift, 13045-755 Campinas, SP, Brazil; ^2^School of Medicine, Federal University of Tocantins, Avenida NS-15, Quadra 109, Norte, S/n-Plano Diretor Norte, Palmas TO 77001-090, Brazil

## Abstract

Synovial sarcoma (SS) is a rare malignant mesenchymal tumor that mainly occurs in body extremities, being uncommon in the head and neck region. In the present study, we described a case of primary intraosseous SS arising in the mandible of a 22-year-old young male. The patient reported a painful swelling on the left side of the mandible for the last 7 months. Imaging exams showed the presence of an expansive and multilocular radiolucent lesion, extending from the left condyle to the mandibular body. The clinic diagnostic hypotheses were ameloblastoma or malignant neoplasm. Histologically, the lesion was characterized by a proliferation of spindle cells exhibiting vesicular nuclei and evident nucleolus. Neoplastic cells were positive for AE1/AE3, cytokeratin 7, vimentin, CD-99, and TLE-1 and negative for CD-34, S-100, SMA, and HHF-35. A combination of clinical, histologic, and immunohistochemical characteristics supported the diagnosis of SS. The patient was referred for treatment, and preoperative exams did not reveal any other tumor foci in the body of the patient. The final diagnosis was of a primary intraosseous SS of the mandible.

## 1. Introduction

Synovial sarcoma (SS) is a rare neoplasm accounting for up 10% of all sarcomas [1–3]. The term SS was first proposed by Knox in 1936 due to the similarity of this neoplasm with normal synovial tissues [4]. Despite the name, this neoplasm does not originate from the synovium or synovial structures. In fact, some studies have suggested that SS can be derived from undifferentiated cells, neural crest stem cells, or pluripotent mesenchymal cells and may arise in any part of the human body [5–8]. Histologically, SS can be classified into biphasic, monophasic, and poorly differentiated. Biphasic SS is composed of spindle cells and epithelial cells, the latter forming nests and glandular structures. Monophasic SS is characterized by highly cellular solid sheets of small spindle cells. Poorly differentiated SS is composed mainly of solid sheets of rounded cells [9–11].

SS usually occurs in extremities, being very rare in the head and neck region [12–14]. In such place, SS often arises in the hypopharynx and parapharyngeal spaces, mainly in the paravertebral connective tissue and being less common in the larynx [15]. In oral cavity, the first case of SS was reported in the base of the tongue of a 23-year-old female [16]. Besides the tongue [17–19], SS can arise in other oral structures such as the buccal mucosa [20, 21], soft and hard palate [22–24], gingiva [25], retromolar area [26], and the floor of the mouth [27, 28]. The involvement of the jaws by SS is extremely rare, and only 23 cases of primary intraosseous SS have been reported in the literature [29]. In this region, SS is usually diagnosed in young female patients, with the mandible and maxilla being equally affected [29]. This article was aimed at reporting a new case of primary intraosseous SS arising in the mandible of a young male patient.

## 2. Case Report

**Figure 1:**
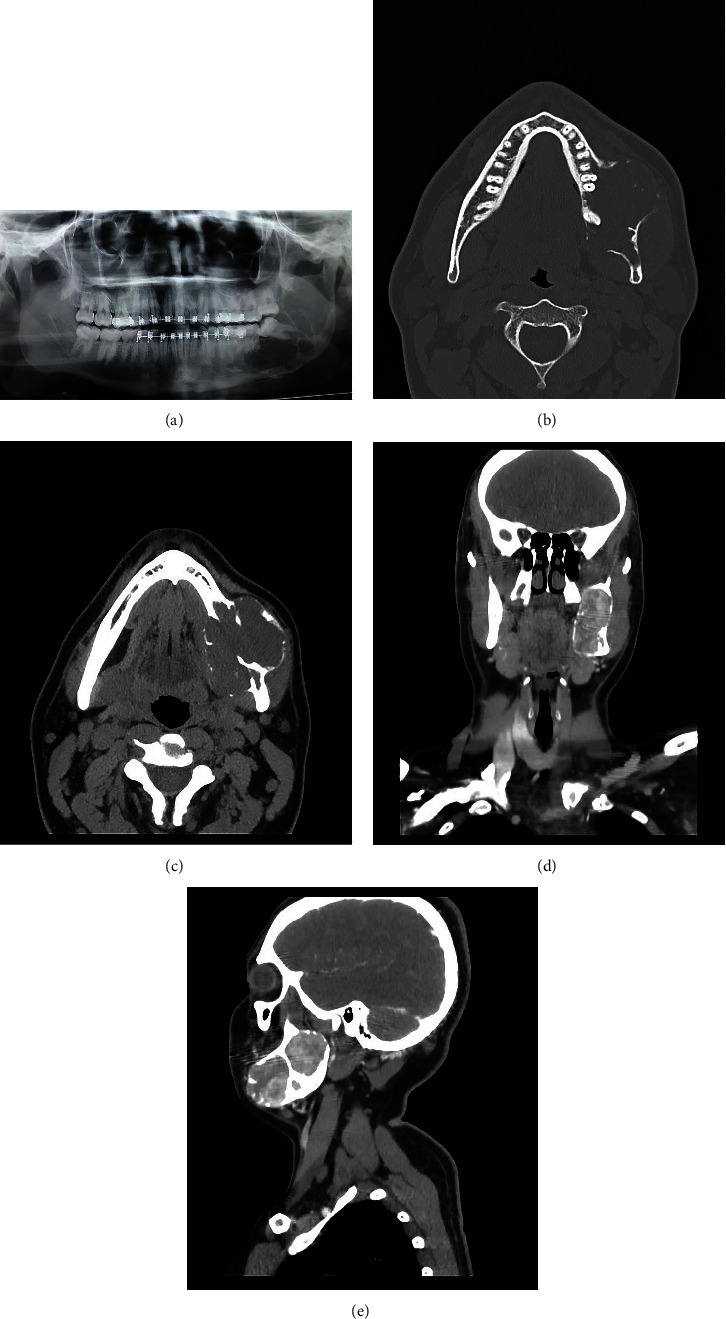
Panoramic radiograph revealed a multilocular radiolucency in the body and ramus of the mandible (a). Computed axial tomography revealed a large hypodense lesion exhibiting cortical bone destruction (b–e).

A 22-year-old male attended the dental emergency service in Palmas (Tocantins, Brazil), reporting pain in the region of the left mandibular body and ramus, which he believed to be related to an unerupted third molar. A panoramic radiographic exam showed the presence of an expansive and multilocular radiolucent lesion, extending from the left condyle to the mandibular body (Figure 1(a)). Computed tomography revealed a hypodense area with cortical bone destruction (Figures 1(b)–1(e)).

The patient was referred to a specialized dental service for evaluation and treatment. In the intraoral examination, an expansion of the cortical bone was noticed in the vestibular and lingual regions of the left mandible. An incisional biopsy was performed, and, during this surgical procedure, a solid, purple-colored lesion with hard consistency was detected near to the cortical bone, while a soft consistency lesion was noticed in the cancellous bone region. The clinic diagnostic hypotheses were ameloblastoma or malignant neoplasm.

**Figure 2:**
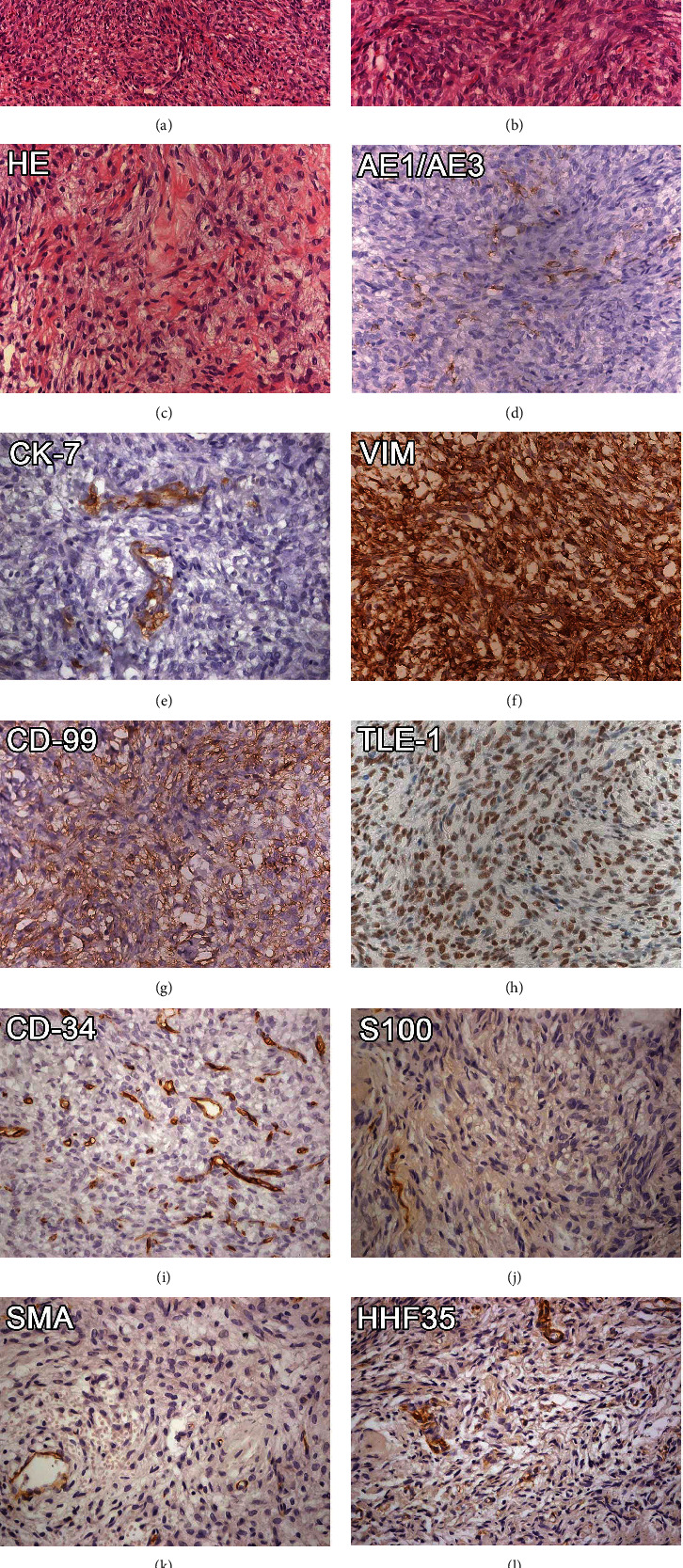
HE staining revealed a neoplasm composed of sheets of spindle cells arranged in bundles and exhibiting a storiform pattern (a, b). Hyalinized collagen and myxoid areas were identified in some parts of the specimen (c). The neoplastic cells were positive for AE1/AE3 (d), CK-7 (e), and CD99 (g); diffusely positive for vimentin (f) and TLE-1 (h); and negative for CD34 (i), S100 (j), SMA (k), and HHF35 (l). Scale bar: A = 200 *μ*m and B − L = 50 *μ*m.

The collected material was fixed in 10% buffered formalin. Paraffin sections were prepared for light microscopy using routine procedures. The sections were stained with hematoxylin and eosin (HE). Microscopic evaluation revealed a hypercellular tumor characterized by a sheet of spindle cells arranged in bundles, exhibiting sometimes a storiform pattern. Individually, the cells showed no defined limits with oval nuclei and loose chromatin (Figures 2(a) and 2(b)). Mitoses or necrosis was not observed throughout the specimen. Tumor stroma was scarce and well vascularized. In focal areas, hyalinized collagen and myxoid areas were identified (Figure 2(c)). Based on microscopic features, a provided diagnosis of sarcoma was made and an immunohistochemistry panel containing vimentin, AE1/AE3, S100, CD-34, CD-99, smooth muscle actin (SMA), and HHF35 was requested. The results showed that neoplastic cells were positive for vimentin and CD-99 (Figures 2(f) and 2(g), respectively). In focal areas of the tumor, the cells were positive for AE1/AE3 (Figure 2(d)), while SMA and HHF35 staining was negative for tumor cells (Figures 2(k) and 2(l), respectively). The positivity for AE1/AE3, in focal areas, leads us to perform additional immunostains for cytokeratin-7 (CK-7) and transducing-like enhancer of split 1 (TLE-1). The tumoral cells showed focal expression of CK-7 (Figure 2(e)). TLE-1 was detected in nuclei of almost all tumoral cells (Figure 2(h)). Taken together, these results rendered the diagnosis of monophasic SS.

The patient was referred to the Head and Neck Medical Service for evaluation and treatment. The patient was submitted to computed tomography of the chest and abdomen and bone scintigraphy. These exams did not reveal any other tumor foci in the body of the patient, and the final diagnosis was a primary SS of the mandible.

## 3. Discussion

Primary intraosseous SS is a rare neoplasm that can arise in the jaws. The cases of SS reported in the literature, in these particular anatomic sites, were commonly detected in young female patients, with the mandible and maxilla being equally affected by SS [29]. In the present manuscript, we reported a new case of primary intraosseous SS exhibiting a monophasic histologic type in the mandible of a young male patient.

SS can be classified histologically as biphasic, monophasic, and poorly differentiated. Biphasic SS is commonly characterized by the presence of glandular structures lined by well-differentiated cuboidal to columnar epithelium, which is surrounded by fibroblast-like spindle cells. Monophasic SS is marked by spindle cells arranged in fascicles exhibiting an ill-defined cytoplasm in a variably collagenous stroma. Poorly differentiated SS is characterized by sheets of uniform, closely packed, rounded cells [30, 31].

This malignant neoplasm contains a translocation t(X; 18)(SS18-SSX1-2), which can be detected by some molecular techniques such as fluorescence *in situ* hybridization [32–34]. The identification of this translocation has been claimed as the gold standard for the diagnosis of SS. Unfortunately, this exam is not always available in laboratories specialized in oral pathology and/or affordable for the patients. In this scenario, an adequate immunohistochemistry panel can be helpful for the diagnosis of this tumor.

In the present case report, the first microscopic impression of the tumor, associated with radiographic aspects, suggested that this neoplasm could be a sarcoma, thus eliminating the possibility of odontogenic tumors, such as odontogenic fibroma, ameloblastic fibroma, and ameloblastic fibrosarcoma. Immunohistochemistry analysis revealed that tumor cells were diffusely positive for vimentin and TLE-1, with scattered cells positive for keratin, especially CK-7, and negative for S100, CD-34, SMA, and HHF35. This panel of antibodies was important to exclude other sarcomas. Thus, the fact that neoplastic cells were negative for specific markers was fundamental for the exclusion of neoplasms derived from nervous (S100), endothelial (CD-34), and muscle tissues (SMA and HHF35). On the other hand, the presence of vimentin and keratin could represent a carcinosarcoma, excluding other malignant spindle cell neoplasms. The positivity for TLE-1, however, provides strong evidence in favor of the diagnosis of SS.

TLE-1 is a member of a large family of proteins that act as corepressor for many transcription factors and plays an essential role in osteogenesis, hematopoiesis, myogenesis, neuronal differentiation, and terminal epithelial differentiation [35–39]. TLE proteins are also effectors of several signaling pathways that control cell fate, such as Notch, Wnt/*β*-catenin, and NF-*κ*B [39–41]. Therefore, the overexpression of these proteins is associated with tumorigenesis in several neoplasms. In SS, TLE-1 can modulate the Wnt/*β*-catenin expression, a well-known signaling pathway involved with the development of this tumor [42–44].

El Beaino et al. in their systematic review and meta-analysis regarding the diagnostic value of TLE-1 in SS highlighted this protein as a legitimate biomarker for SS [45]. Despite the fact that hybridization analysis for the chromosomal t(X; 18) translocation was considered the most relevant exam for the diagnosis of SS, the pathologist, however, must be aware that diagnosis of SS should be also based on clinical context, histologic features, and immunohistochemistry profile [11], once some tumors can exhibit other types of genetic mutations [45–48]. Indeed, molecular testing is not required if the diagnosis of SS was certain or probable on the basis of clinical, histologic, and immunohistochemical evaluation [49].

As described above, the monophasic pattern of SS is very a challenging diagnosis especially in oral pathology, where this tumor is very rare. In our case, the association of clinical history, microscopic morphology features, and immunohistochemistry profile leads us to the final diagnosis of SS.

SS has an aggressive behavior, and, for this reason, wide surgical excision, to obtain clear margins, followed by adjuvant radio and/or chemotherapy, is the recommended treatment modality [50, 51]. Primary intraosseous SS of the jaws has a high incidence of local recurrence and tumor-related death [29]. Thus, long-term follow-up is necessary and important for early detection of recurrence. In our reported case, the patient has been followed up for the last two years with no sign of the disease.
